# Biological Activities of Fucoidan and the Factors Mediating Its Therapeutic Effects: A Review of Recent Studies

**DOI:** 10.3390/md17030183

**Published:** 2019-03-20

**Authors:** Yu Wang, Maochen Xing, Qi Cao, Aiguo Ji, Hao Liang, Shuliang Song

**Affiliations:** 1 Marine College, Shandong University, Weihai 264209, China; wy392191187@163.com (Y.W.); sddxxmc@163.com (M.X.); sddxcqq@163.com (Q.C.); jiaiguo@sdu.edu.cn (A.J.); lianghao@sdu.edu.cn (H.L.); 2 School of Pharmaceutical Sciences, Shandong University, Jinan 250012, China

**Keywords:** acid polysaccharide, fucoidan, biological structure, biological function

## Abstract

The marine acid polysaccharide fucoidan has attracted attention from both the food and pharmaceutical industries due to its promising therapeutic effects. Fucoidan is a polysaccharide that mainly consists of L-fucose and sulphate groups. Its excellent biological function is attributed to its unique biological structure. Classical activities include antitumor, antioxidant, anticoagulant, antithrombotic, immunoregulatory, antiviral and anti-inflammatory effects. More recently, fucoidan has been shown to alleviate metabolic syndrome, protect the gastrointestinal tract, benefit angiogenesis and bone health. This review focuses on the progress in our understanding of the biological activities of fucoidan, highlighting its benefits for the treatment of human disease. We hope that this review can provide some theoretical basis and inspiration for the product development of fucoidan.

## 1. Introduction

Advances in biopharmaceuticals and genetic engineering approaches have led to widespread interest in the therapeutic benefits of polysaccharide bio-macromolecules, particularly acidic polysaccharides from marine sources. Amongst these, fucoidan has shown promise. Fucoidan was first discovered in 1913. Since then, research on fucoidan has become more widespread; in the past 5 to 10 years, the number of papers on fucoidan has increased significantly [1]. Brown seaweeds containing fucoidan have become part of some Asian diets, especially Japan, China and South Korea.

Fucoidan is a complex sulfated polysaccharide derived from the cell walls of brown seaweeds and some marine invertebrate tissues [2,3]. Fucoidan is a water-soluble heteropolysaccharide composed of L-fucose and sulphate groups, the main monosaccharide component of which is L-fucose-4-sulfate. It also consists of other monosaccharides including uronic acid, galactose, xylose, mannose, rhamnose, glucose, arabinose and xylose [4,5]. Fucoidan is composed of two chain structures, one with (1→3)-α-L- fucopyranose as the chain and the other with α-L-fucopyranose linked by (1→3) and (1→4) as the main chain. Single and double substitutions in the sulphate groups at the C-2 or C-4 positions of both skeletons can occur. Some fucoidans possess substituted branches at the C-2 and C-3 positions [6,7]. Generally, the composition and structure of fucoidan is dependent on seaweed species, geographic location, harvesting season, anatomical regions, and the extraction procedures [8,9,10,11,12,13,14]. Fucoidans from different natural resources are typically extracted in a water bath, acid bath, or through microwave heating. The bioactivity of fucoidan varies according to the extraction method and its molecular weight (Mw) can vary from 10,000 to 100,000 Da depending on the sample source [1,15,16,17]. Fucoidan is non-toxic, rarely causes irritation reactions, and has many biological activities that are beneficial to therapeutic applications [18]. Due to the continual development of marine natural products, fucoidan has emerged as a research hotspot regarding its separation, purification, preparation, structural analysis, bioactivity, and oral absorption.

**Figure 1:**
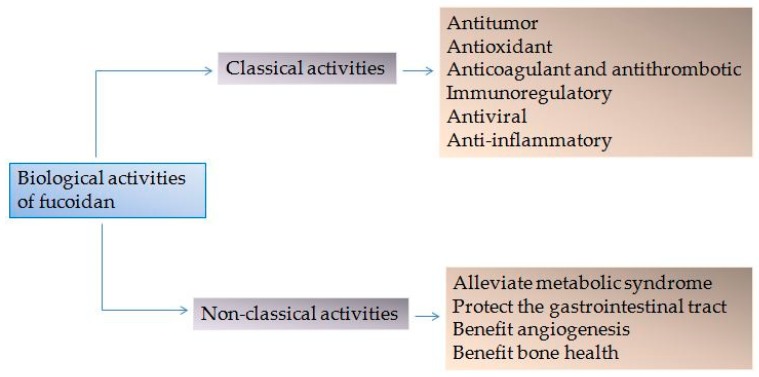
Biological activities of fucoidan.

In this review, we focus on progress regarding the “classic” bioactivity of fucoidan, and it’s newly identified cellular functions. An array of studies has reported its antitumor, antioxidant, anticoagulant, antithrombotic, immunoregulatory, antiviral and anti-inflammatory effects. More recently, novel biological activities of fucoidan, as outlined in Figure 1, have been discovered based on its effects on these “classical” pathways [19,20,21,22,23]. We further highlight important factors influencing the bioactivity of fucoidan, including Mw, sulfate groups, sources, and extraction methods. We herein provide a theoretical basis for further development and utilization of fucoidan.

## 2. Classical Activities of Fucoidan

### 2.1. Antitumor Activity

Cancer remains a major global cause of morbidity and mortality [24]. Chemotherapy is commonly used in cancer treatment, but side-effects including nausea and bone marrow failure are common, as is multidrug resistance [25]. New and effective antitumor compounds with low toxicity are urgently required. When isolated from natural products (particularly marine resources), fucoidan displays significant antitumor activity against liver, gastric, cervical, lung and breast cancers [21,26,27,28,29,30]. The antitumor mechanisms are mediated through its ability to induce apoptosis in tumor cells, inhibit tumor angiogenesis, potentiate the toxic effects of traditional chemotherapy drugs, and enhance immune function [27,31,32,33,34,35]. These mechanisms are summarized in Table 1. At the same time, Table 1 also summarizes the signaling pathways involved in the anti-cancer effect of fucoidan, which is instructive for later research. It is worth noting, however, that the cancer cell lines typically have abnormal signal transduction pathways and are therefore not suitable for assessing cellular signaling to some extent. The induction of apoptosis in cancer cells is ubiquitous and mediated through deoxyribonucleic acid (DNA) damage, leading to chromosome condensation. Low Mw fucoidan (LMWF) triggers G1 arrest and apoptosis in human colon cancer cells (HCT116 cells) via a p53-independent mechanism [36]. Fucoidan was also found to induce autophagy in human stomach cancer cells (AGS cells) assessed through the transition of microtubule-associated protein light chain 3-I into light chain 3-II, and the accumulation of beclin-1 [37]. Autophagy induction leads to apoptosis in treated AGS cells.

The antitumor activity of fucoidans are defined by their chemical structure, including the content of sulfate groups, Mw, the ratio of monosaccharides, and binding of sugar residues [7]. Yang et al. compared the antitumor activity of natural fucoidan extracted from the spore leaves of *Undaria pinnatifida* (*U. pinnatifida*, 5100 kDa) and fucoidan that was depolymerized in a boiling water bath (490 kDa). No significant difference in the sulfate content of the two fucoidans was observed, but the antitumor activity of LMWF was over 2-fold higher at the same concentrations [38]. In general, over-sulfated fucoidan had stronger antitumor activity than natural fucoidan [39]. But interestingly, it has been reported that the antitumor activity of purified fucoidan was not correlated with its sulfate content. Fucoidan isolated from *Sargassum hornery* has no sulfate groups but its antitumor activity is comparable to that of fucoidan with a 16.9% sulfate content [40]. The cause of this phenomenon may be due to the different sources, purity, and the different tumor models assessed. Although the antitumor activity of fucoidan is determined by its biological structure, it is not just a single factor. The antitumor activity of the tested fucoidan is determined not only by the amount of sulfate groups, but by a combination of factors such as monosaccharide residues ratio, type of sugar residues bounding and so on.

### 2.2. Antioxidant Activity

Reactive oxygen species (ROS) include superoxide anion, hydroxyl radical, hydrogen peroxide, singlet oxygen, and nitric oxide (NO) [52]. In general, low ROS levels regulate many biochemical processes that are required for cell division; whilst excessively high levels of ROS disrupt redox homeostasis damaging lipids, proteins, and nucleic acids, leading to various physiological diseases such as cancer, coronary heart disease, atherosclerosis, diabetes, neurodegenerative diseases, inflammatory diseases, and aging-related diseases [53,54,55]. Antioxidants protect the body from ROS. Previously described antioxidants include butyl hydroxyanisole, butylated hydroxytoluene and tertiary butyl hydroquinone, but these compounds are toxic and may be carcinogenic [56]. The identification of non-toxic antioxidant compounds is an area of intense research.

As a natural active polysaccharide, fucoidan is a known ROS scavenger. It was reported that fucoidan extracted from *Laminaria japonica* (*L. japonica*) can be used to treat diseases caused by free radical damage due to its antioxidant properties [4,16]. Similarly, fucoidan extracted from *U. pinnatifida* exhibits significant antioxidant activity [12]. It was shown that fucoidan can reduce the accumulation of amyloid-β and ROS to inhibit amyloid-β-induced toxicity [57]. Superoxide dismutase and glutathione activity were also induced following fucoidan treatment [58]. Accordingly, fucoidan is widely used as a natural antioxidant polysaccharide in skin care products such as neuro-health foods.

Several factors determine the antioxidant activity of fucoidan, including concentration, Mw, the degree of sulphation, substitution groups and their positions, type of sugar, and glycosidation branching [59,60,61]. It has been shown that components isolated from brown algae exhibit ROS scavenging activity in a concentration-dependent manner [62]. Furthermore, Mw significantly influences the hydroxyl radical scavenging activity and reducing ability of fucoidan [63]. High Mw crude fucoidan may be difficult to cross the lipid bilayer and exert its biological activity, whilst LMWF and its derivatives have high antioxidant capacity [64]. The low Mw sulfated polysaccharide obtained from *L. japonica* had stronger effects on the oxidation of low density lipoprotein compared to crude fucoidan [65]. These results indicate that a lower Mw may be beneficial for antioxidant activity [66].

Most importantly, the substituents of fucoidan play a major role in its antioxidant activity [62,67]. Wang et al. studied the antioxidant mechanisms of LMWF and identified an influence of the substituent groups [67]. In general, electron withdrawing groups, which enhance the antioxidant ability of LMWF, change the polarity of the compound or activate hydrogen atoms of anomeric carbons. Cations such as amino groups act weakly during oxidation resistance as they cannot activate a hydrogen atom. Groups substituted at different positions also influence antioxidant activity. A positive correlation between sulfate content and antioxidant capability has been reported. Moreover, the ratio of sulfate content and fucose influences hydroxyl radical scavenging ability [4,5]. High fucose and sulfate content were shown to significantly influence the ablation of lipid accumulation by fucoidan [68]. It is not surprising that the factors determining the antioxidant activity of fucoidan are comprehensive and not a single factor. The content and location of the sulfate groups which affected by the extraction method are the determining factors. As such, the extraction method influences antioxidant activity. In this regard, fucoidan isolated through microwave assisted extraction technology at 90 °C has the highest antioxidant capability [9].

Taken together, this body of evidence demonstrates that the antioxidant mechanism of fucoidan has not been completely elucidated. Chemical modifications to fucoidan can improve its antioxidant activity, holding promise for its use in ROS-related disease.

### 2.3. Anticoagulant and Antithrombotic Activity

One of the treatment options for disseminated intravascular coagulation and venous thromboembolic disease is the use of anticoagulants [69]. Heparin is one of the most widely used intravenous anticoagulants due to its unique polyanionic properties. However, heparin is limited by its hemorrhagic effects and the occurrence of heparin-induced thrombocytopenia [70]. Alternative drugs with fewer side effects are thus required, and safer and more effective anticoagulants are under development [71]. Fucoidan has significant anticoagulant effects, which is the most studied but these are complex and structure dependent [72]. Fucoidan is an effective thrombin and factor Xa inhibitor, with anti-thrombin effects mediated through heparin cofactor II, and other internal and external coagulation factors [73,74]. Studies have shown that fucoidan and its derivatives (including sulfated, phosphorylated and aminated fucoidan) can prolong partial thromboplastin time and thrombin time. Fucoidan inhibits the endogenous coagulation cascade, thrombin activity, and/or fibrin polymerization, but its derivatives affect the determination of prothrombin time, and the extrinsic coagulation cascade. This high level of anticoagulant activity is mainly attributed to the formation of ternary complexes with antithrombin III- Xa and antithrombin III- Xa, and direct binding to thrombin [75]. Thus, fucoidan shows promise as a new, effective, non-toxic anticoagulant, with fewer side effects.

Evidence indicates that the anticoagulant activity of fucoidan strongly depends on its Mw, degree of sulfation, sulfation mode, sulfate/total sugar ratio, and glycoside branching [76]. Fucoidan and its derivatives have higher anticoagulant activity than LMWF, indicating that Mw and conformation are contributing factors. Chandía et al. prepared LMWF by free radical depolymerization and displayed good anticoagulant activity [77]. Jin et al. found that the average Mw and galactose content affected the anticoagulant activity of fucoidan in a dose-dependent manner [78]. According to previous reports, fucoidans with an Mw of 50–100,000 Da are potential anticoagulants, whilst fractions >850,000 Da Mw lack anticoagulant activity [79]. Fucoidan polymers were shown to exhibit the strongest anticoagulant activity with Mws ranging from 10 kDa to 300 kDa [38]. Studies have found that fucoidan of high sulfate and low uronic acid content displays high anticoagulant activity [73,79]. The LMWF from *L. japonica* was separated into three components and it was found that the higher the content of sulfated groups, the higher the anticoagulant activity [5]. However, the anticoagulant activity of purified fucoidan from the brown Seaweed *Canistrocarpus cervicornis* did not correlate to sulfate content. Undoubtedly, this is related to the position of the substituent. Progress has been made in the study of sulfate factors. Anticoagulant activity was greatly enhanced when fucoidan was sulfated at the 2-O and 3-O- positions of the fucopyranose residue [80].

The antithrombotic activity of fucoidan is largely attributed to its anticoagulant activity. In addition, fucoidan has affinity for P-selectin that is expressed by activated platelets in the thrombus, enhancing the risk of bleeding if injected with recombinant tissue plasminogen activator due to thrombolysis [81]. Previous studies have confirmed the antithrombotic activity of fucoidan from the brown seaweed *Saccharina latissimi* [75]. The Mw of fucoidan from *Ecklonia kurome* increased from 10 kDa to 50 kDa as did the antithrombotic activity [82].

### 2.4. Immunoregulatory Activity

The immunoregulatory activity of fucoidan is a hot research topic. It is generally believed that the major mechanism by which fucoidan protects cells is through the activation of the host immune responses. The various beneficial pharmacological effects of fucoidan, such as antiviral and antitumor activity, are attributed to its ability to modulate cellular immune function. It is speculated that fucoidan binds to different receptors such as Toll-like receptors (TLRs) on dendritic cells (DCs), macrophages and other monocytes, and then activates them to release pro-inflammatory factors, cytokines and chemokines, which can help the host to form a strong immune response and achieve multi-channel and multi-level regulation of the immune system [83,84]. Numerous studies have validated the effects of fucoidan on immune regulation. It has been reported that fucoidan can be used as an adjuvant by inducing the up-regulation of CD40, CD80 and CD86 expression and production of interleukin-6 (IL-6), IL-12 and tumor necrosis factor-α (TNF-α) in spleen cDCs, which may have an effect on the development of tumor vaccines. Moreover, fucoidan can enhance a variety of beneficial effects of lactic acid bacteria on immune functions by improving the Th1/Th2 immune balance [85]. It was shown that through its ability to regulate the immune response and reduce inflammation, fucoidan can treat gastrointestinal ulcers caused by oral aspirin [86]. The fucoidan extracted from Japan *F. vesiculosus* can promote TNF-α and IL-6 production in peritoneal macrophages [87].

There has also been considerable progress in our knowledge of the regulation of immunomodulatory signaling. Fucoidan regulates P38 mitogen-activated protein kinase (p38MAPK) and nuclear factor-kappa B (NF-κB-dependent) signaling to promote NO release from RAW264.7 macrophages [88]. Similarly, the LMWF (<10 kDa) prepared from New Zealand *U. pinnatifida* significantly activated RAW264.7 macrophages through identical signaling pathway to produce NO, TNF-α and IL-6 [89]. In terms of immune regulation, the structure-activity relationships of fucoidan remain controversial. Khil’chenko et al. demonstrated that the presence of sulfate and acetyl groups had a major impact on the immunomodulatory activity of fucoidan, which could reduce the release of inflammatory cytokines [87]. These studies laid a theoretical foundation for the development of fucoidan as a new generation of polysaccharide immunomodulator. Similarly, the immunological activity of fucoidan contributes to tumor therapy. As such, the discovery and evaluation of fucoidan regarding its immunostimulatory properties has emerged as an important research direction in chemistry, biology, and medical fields.

### 2.5. Antiviral Activity

Viral infections are common and a massive burden to healthcare systems across the globe. Specific antiviral drugs for a range of viral infections are lacking and systemic supportive therapy and symptomatic treatment remain the mainstays. Nucleoside drugs were the earliest clinically used antivirals, but the side-effects associated with these drugs, including acute renal failure, increase with increasing drug doses [90]. New and effective agents are therefore urgently required. In recent decades, Chinese herbal medicines including polysaccharides have been shown to alleviate the symptoms of specific viral diseases and shorten the disease period [91]. In the search for natural antiviral agents with carbohydrate properties, studies have focused on sulfated polysaccharides. It was reported that fucoidan has broad spectrum antiviral activity and low toxicity and resistance [92]. In general, fucoidan inhibits human immunodeficiency virus, herpes simplex virus (HSV), human cytomegalovirus, influenza virus and bovine viral diarrhea virus [93,94,95,96,97,98,99,100]. The mechanism of antiviral activity is to inhibit viral adsorption onto cells, thereby impeding virus entry [93,96,101]. The sulfated polysaccharide inhibits virus attachment to host cells by interacting with the positively charged domain of viral envelope glycoproteins involved in virus attachment [97,102]. It has also been reported that the antiviral activity of fucoidan is achieved through the phagocytic function of immune cells, and humoral immunity. LMWF from *L. japonica* can improve the function of immune organs and increase thymus index and spleen index. The effects on the phagocytic index and the phagocytic coefficient also increase with increasing dose. Moreover, LMWF can increase the half hemolysin value in middle and high doses [103]. Fucoidan isolated from *Dndaria pinnatifida* was shown to effective against HSV-1 by directly inhibiting viral replication and/or stimulating innate and adaptive immune defenses [104].

The antiviral activity of sulfated polysaccharides such as fucoidan are primarily dependent on structural characteristics such as the degree of sulfation, sulfate group content, Mw, monosaccharide composition, molecular conformation and stereochemistry [105,106]. The key determinants are sulfate content and Mw. The antiviral activity of sulfated polysaccharides has been reported to increase with Mw and sulfate content [107]. A sulfate at C-4 of the (1–3)-linked fucopyranosyl unit appears important for the anti-HSV activity of fucoidan [93].

Polyanions exhibit potent antiviral activity in vitro, but in vivo, the low bioavailability of the drugs limits their applications [108]. As a polyanionic drug, fucoidan may be similar. Hence, fucoidan must be used in combination with a drug delivery system to exert a higher therapeutic effect. In summary, fucoidan has broad medicinal prospects as an effective, low-toxic antiviral compound.

### 2.6. Anti-Inflammatory Activity

The inflammatory response is a reflection of the body’s resistance to inflammatory factors. Inflammation is a double-edged sword when overactive causes tissue damage, but when correctly regulated, mediates the body’s resistance to disease. During the inflammatory response, the release of NO increases as does the expression of inflammatory factors. Numerous diseases of the human body are accompanied by inflammatory reactions including atherosclerosis, diabetes, neurodegenerative diseases, inflammatory diseases, chronic autoimmune diseases and diseases related to aging. Experimental evidence indicates that fucoidan from marine resources regulates the inflammatory response, particularly the intestinal inflammatory response. The role of fucoidan in the treatment of intestinal inflammation is detailed in Section 3.2.

Fucoidan can significantly inhibit the release of NO induced by bacterial lipopolysaccharide, and reduce inflammation. Fucoidan is also a ligand for macrophage scavenger receptor A, which can be taken up by macrophages and inhibits NO production. Inhibiting the migration of leukocytes to inflammatory tissues is another manifestation of the anti-inflammatory activity of fucoidan [109]. It was reported that fucoidan from wakame reduces the expression of cyclo-oxygen-ase-2 in rabbit articular chondrocytes in a dose- and time-dependent manner, thereby exerting anti-arthritis effects [110]. Notably, fucoidan can influence the inflammatory response in patients with advanced cancer [111]. In addition, fucoidan can synergistically enhance the efficacy of anti-inflammatory drugs. Fucoidan-coated ciprofloxacin-loaded chitosan nanoparticles can effectively treat intracellular and biofilm infections of Salmonella [112]. Compared to other small molecule anti-inflammatory drugs, fucoidan as a macromolecular substance has broad prospects as a direct drug or food adjuvant.

## 3. Non-Classical Activities

### 3.1. Angiogenesis

Angiogenesis is important in reproductive, developmental, and repair processes [113]. Angiogenesis is a complex process in which pro-angiogenic factors and inhibitors coordinate with each other. When this balance is destroyed, the vascular system’s ability to repair degenerate blood vessels is impaired. This requires both anti-angiogenic and pro-angiogenic drugs for treatment. Angiogenesis involves the proliferation, differentiation and migration of mature endothelial cells, and is regulated by various endothelial angiogenic factors, including platelet-derived growth factor, vascular endothelial growth factor (VEGF) and fibroblast growth factor (FGF) [114]. One of the mechanisms of action of fucoidan in the treatment of tumors is to inhibit angiogenesis. Anti-angiogenic drugs have become increasingly popular as vascular-targeted drugs improve the microenvironment of the tumor. Fucoidan inhibits angiogenesis through regulation of the expression of VEGF and endothelial cell plasminogen activator inhibitor-1. At the same time, the regulation of matrix metalloproteinases (MMPs) and chemokine CXCL12 can inhibit cell migration. Persulfated fucoidan can alleviate the destruction of the basement membrane and cell migration, and inhibit the angiogenesis of HUVECs on matrigel [115]. Fucoidan isolated from the brown seaweed *Sargassum fusiforme* can inhibit the angiogenesis of human microvascular endothelial cells in a dose-dependent manner [116]. The major signaling pathways involved in the inhibition of angiogenesis are shown in Table 1.

Fucoidan also promotes angiogenic activity. Fucoidan has pro-angiogenic properties through its ability to modulate heparin-binding growth factors such as FGF-2. At an antithrombotic concentration, fucoidan enhances FGF-2-induced angiogenesis by regulating the expression of angiogenic surface proteins (mainly α6) [117]. LMWF promotes FGF-2-induced angiogenesis in human endothelial cells, which may be beneficial for blood remodeling in ischemic areas [118]. Recent studies have reported that fucoidan activates p38 and C-Jun N-terminal kinase (JNK) by targeting the AKT/MMP-2 signaling axis in HUVECs [114].

Undoubtedly, structural factors, particularly Mw, influences the anti-angiogenic and pro-angiogenic activities of fucoidan. It has been reported that natural fucoidan with an Mw of 47.5 kDa has a certain degree of sulfation (20.8%) and significantly inhibits angiogenesis. A derivative of F2MDI with an Mw of 12.4 kDa and a sulfate content of 7.5% showed reduced inhibition [116]. Fucoidan isolated from the marine brown alga *L. japonica* has an Mw of 30 kDa and a sulfate content of 33.2%. This fucoidan displays significant angiogenesis inhibition in vitro, while an Mw of 15–20 kDa and a low degree of sulfation (8.2%) does not inhibit HUVEC tube formation [119]. These results indicate that Mw and sulfation play key roles in the anti-angiogenic activity of fucoidan. In general, 20–30 kDa is the critical Mw.

### 3.2. Treatment of Intestinal Diseases

Inflammatory bowel disease (IBD) is an idiopathic intestinal inflammatory condition involving the ileum, rectum, and colon. The healing of intestinal inflammation is a complex process due to the presence of intestinal microbes. One such factor in the pathogenesis of IBD is the oxidative stress-induced disruption of intestinal epithelial cells and subsequent increases in paracellular permeability. In recent years, the pharmacological activity of fucoidan in IBD was discovered. Zhang et al. revealed that fucoidan can reduce mucosal damage and crypt destruction in the colon of dextran sodium sulfate-treated mice to treat chronic colitis [120]. Fucoidan from *Cladosiphon okamuranus Tokida* improved chronic colitis by downregulating the expression of the proinflammatory cytokine IL-6 in the colonic epithelial cells of IBD mice [121]. It was shown that fucoidan benefitted IBD by upregulating the expression of the tight junction protein claudin-1 to protect epithelial barrier function from oxidative damage [122]. Moreover, Tanoue et al. established a co-culture system with intestinal epithelial Caco-2 cells (apical side) and macrophage RAW264.7 cells (basal side) to simulate intestinal inflammation in vivo. They found that fucoidan from brown algae can suppress IL-8 gene expression in this co-culture system [123].

A growing body of experimental evidence indicates that fucoidan plays an important role in the prevention of gastric ulcers [18]. Fucoidan reduces the expression of IL-10 and increases the levels of IL-6 and interferon-gamma to prevent aspirin-induced gastric ulcer [124]. In addition, fucoidan from *Cladosiphon okamuranus* benefits the treatment of gastric ulcers by blocking the Le (b)—and the sulfatide-mediated adhesion of *Helicobacter pylori* to gastric cells [125]. The mechanisms to prevent ethanol-induced gastric ulcer formation typically includes anti-oxidant and anti-inflammatory signaling, and the regulation of mitogen-activated protein kinases and matrix metalloproteinases [126,127]. Variations in the protective effects of the gastrointestinal tract of different fucoidans have also been reported, attributed to differences in Mw and chain conformation [128].

With the advent of high-throughput sequencing technology and bioinformatics, the interaction between fucoidan and intestinal microflora has become an intense research focus. The human intestinal tract is densely composed of intestinal microflora that regulate human immunity, digestion, metabolism, and disease. The effects of fucoidan on the intestinal tract are mediated through its interaction with intestinal microbes. Studies suggest that fucoidan from Pearsonothuria graeffei (fuc-Pg) can alleviate the dysregulation of intestinal microflora in high fat diet (HFD) mice by increasing the abundance of benign microorganisms [129].

### 3.3. Treatment of Metabolic Syndrome

Metabolic syndrome (MetS) generally refers to the pathological state in which proteins, fats, carbohydrates and other substances in the body are metabolically disordered. These disorders are the pathological basis of cardiovascular and cerebrovascular disease and diabetes. MetS typically requires multi-drug treatment, but individual risk factors remain uncontrollable [130]. Natural products that can treat or alleviate MetS are highly attractive and marine polysaccharides have been reported to reduce MetS. Fucoidan can alleviate MetS related disorders, including obesity, hyperlipidemia, hyperglycemia and hypertension through different regulatory mechanisms.

The inhibition of lipogenesis and enhancement of lipolysis makes fucoidan attractive for the treatment of obesity. Fucoidan can down-regulate epididymal expression in adipose tissue in HFD mice, thereby improving obesity [131]. In this case, adipogenic markers and validation-associated cytokines including adipocyte protein 2, peroxisome proliferator-activated receptor-γ, and CCAAR/enhancer binding proteins-αwere reduced [132]. Fucoidan has also been studied in terms of its antihypertensive and lipid-lowering effects. Fucoidan oligosaccharide displays antihypertensive effects in renal vascular hypertensive rats, mediated through its ability to inhibit plasma angiotensin II production. In atherosclerosis models from apoE knockout mice, Xu et al. demonstrated that LMWF can inhibit the inflammatory response and alter lipid metabolism by preventing macrophage development into foam cells and through the inhibition of macrophage migration [133].

Fucoidan has received intense interest as a hypoglycemic agent in diabetes treatment. LMWF can protect diabetic Goto-Kakizaki rats from vascular endothelial function and lower blood pressure [134]. Fucoidan extracted from *F. vesiculosus* is an alpha-glucosidase inhibitor that can be used to treat type 2 diabetes [135]. Fucoidan through its attenuation of VEGF, can also reduce diabetic retinopathy [136]. Modulation of AMPK signaling and KB/GLUT4 activity are the reported mechanisms by which fucoidan improves glucose tolerance [137,138].

With the rapid development of intestinal microbes, it is recognized that fucoidan can act as a prebiotic to regulate the intestinal ecosystem. Promoting the growth of beneficial bacteria represents a mechanism by which fucoidan reduces MetS, but the mechanisms underlying this remain unclear [139]. Parnell et al. reported that prebiotics (which may include fucoidan) can regulate blood sugar and lipid metabolism by stimulating the growth of probiotics [140]. Fuc-Pg can be used as a functional food for the treatment of metabolic syndrome. Fuc-Pg can reduce weight gain in HFD-fed mice, reduce hyperlipidemia, and protect the liver from steatosis. At the same time, fuc-Pg reduces serum inflammatory cytokines and reduces macrophage infiltration into adipose tissue. The biological activity of this treatment for metabolic syndrome is primarily related to the 4-O-sulfated structure of fucoidan [129]. The utilization of fucoidan to treat or ameliorate MetS represents a major direction for the discovery and development of new medicinal compounds. It should, however, be noted that not all fucoidans influence MetS.

### 3.4. Bone Health Supplements

The steady state balance between osteoblasts and osteoclasts contributes to the healthy development of bone tissue. Various diseases including osteoporosis occur when this balance is disrupted, decreasing osteoblast formation and increasing osteoclast absorption [141]. Treatment strategies for osteoporosis often require the use of bone forming and osteoclast inhibiting drugs. Fucoidan holds promise for use as a bone health supplement.

Fucoidan extracted from *Apostichopus japonicus* is a potent inhibitor of osteoclastogenesis [142]. The combination of fucoidan and receptor activator for nuclear factor-κ B ligand (RANKL) or RANK leads to a decrease in osteoclast differentiation. Based on this theory, fucoidan inhibits the osteoclastogenesis of bone marrow macrophages by inhibiting RANKL-induced activation of MAPKs, and through the downregulation of genes involved in osteoclast differentiation and resorption [143]. It is speculated that this effect is promoted by its sulfated region. Studies on the inhibition of osteoclasts are rare, with studies on the induction of osteoblast differentiation more common.

Due to intense research on the induction of osteoblast differentiation, fucoidan was shown to enhance the proliferation of human alveolar bone marrow-derived mesenchymal stem cells (hABM-MSCs), osteoblast MG-63 cells, human adipose-derived mesenchymal stem cells (hADSCs) and mesenchymal cell lineage-human amniotic fluid stem cells [144]. Fucoidan can stimulate alkaline phosphatase, mineral deposition, and bone morphogenetic protein 2 (BMP2), which are associated with bone mineralization in osteoblasts [145,146]. JNK and extracellular signal-related kinase (ERK) signaling, mediated by Smad 1/5/8 and BMP2, mediate the effects of fucoidan on osteoblast differentiation [144]. It was also reported that fucoidan differentially influences angiogenesis when promoting osteoblast differentiation. Fucoidan may reduce blood vessel formation in bone tumors such as osteosarcomas [147]. However, others showed that fucoidan can accelerate the formation of new blood vessels and partially promote bone formation in a rabbit model of skull defects [148]. This difference may lie in cell-cell communication among different cell types, especially the complex interaction between osteoblasts and endothelial cells. In general, different molecule weight of fucoidan exhibits different activities, and it seems that a wide range of molecular weight (3.3–100 kDa) fucoidan can better investigate the therapeutic potential of bone regeneration. The precise molecular mechanisms by which this phenomenon occurs have not been elucidated, but are likely to be related to the fucoidan preparation methods [149].

It should be noted that these studies are limited to in vitro experiments and further in vivo studies are required to verify the potential of fucoidan. Regarding its osteogenic effects, 3D culture systems are predicted to narrow the gap between in vitro studies and clinical trials [150]. Of the in vivo experiments performed, LMWF has been shown to inhibit osteoclast differentiation and bone resorption in ovariectomized Sprague-Dawley rats, demonstrating its potential in the treatment of osteoporosis [151]. The extraction of LMWF from fresh *Sargassum hemiphyllum* also increased bone density and ash weight in C57BL/6J female mice [152]. Based on these findings, we can conclude that fucoidan is a promising osteogenic drug. Further research in this area is now required, and many challenges remain.

### 3.5. Other Activities

In addition to the broad biological activities of fucoidan, more specific effects have recently been reported. Fucoidan from the brown seaweed *Chordaria flagelliformis* is a potent stimulator of hematopoietic function in cyclophosphamide-induced mouse models [153]. This fucoidan was then chemically transformed and modified, and it was found that the fully sulfated synthetic octasaccharide is an effective stimulator of hematopoietic function and can be considered for the development and treatment of immunosuppressive complications [154]. Most importantly, fucoidan has neuroprotective effects and shows promise for the treatment and/or alleviation of senile dementia. Fucoidan can alleviate b-amyloid-induced neurotoxicity in basal forebrain neurons [155]. Moreover, fucoidan can improve ROS production in transgenic Alzheimer’s Disease models [57]. Fucoidan has also been proposed as a contraceptive as it inhibits the binding of sperm to the human zona pellucida [156]. In addition, for the treatment of parasites, fucoidan mediates the leishmania effect by activating host immune responses [157].

## 4. Concluding Remarks and Future Outlooks

Other excellent biological activities of fucoidan are being widely discovered. As an acidic heteropolysaccharide, the biological activities of fucoidan are multi-factorial. Important structural issues of biological activity appear to include the degree of sulfation and Mw of fucoidan. It seems that fucoidan exerts its biological activities through low Mw and sulfate groups. But certainly, there are exceptions. The differences between previous studies are related to the molecular weight and sulfate content of fucoidan, as well as the extraction methods used, the algae source, the time of harvest, and the type of algae. For the majority of fucoidans, the structural skeleton is still unclear and the location and branching sites of the specific sulfate groups are uncharacterized. This creates a level of complexity to fucoidan that makes it difficult to elucidate structure-activity relationships. This review has, to the largest possible extent, elucidated the basic biological activities of fucoidan. At the same time, we comment on important factors mediating its bioactivity, although the structure-function relationship remains controversial. It is hoped that further evidence will emerge in future studies.

To-date, support for the use of fucoidan as a disease-assisted diet supplement is increasing. Although extensive biological activities of fucoidan have been demonstrated with promising preclinical results, its application in clinical practice remain limited. An important barrier for its progression may be the lack of pharmacokinetic data. As a highly polar polysaccharide molecule, fucoidan has a limited ability to pass through intestinal epithelial cells. Oral administration is the simplest method of administration, but due to the Mw of the compound, oral bioavailability is low. In future studies, a clear understanding of technical problems concerning the preparation, quality standards, and administration of fucoidan must be defined to fully harness its clinical potential.

## Figures and Tables

**Table 1:** Antitumor mechanisms of fucoidan.

Mechanism of Action	Sources	Tumor Models	Signaling Pathway (↑: Activation; ↓: Inhibition)	Reference
Tumor cell proliferation/apoptotis induction	*F. vesiculosus* (Sigma)	Human leukemic cells	↑ MAPK pathway	[32]
	*F. vesiculosus* (Sigma)	Human colon cancer HT-29 and HCT116 cells	↑ Death receptor-and mitochondria-mediated apoptotic pathways	[41]
	Species unknown, NPO laboratory	Human breast cancer *MCF-7 cells*	↑ Caspase- dependent pathway	[21]
	*F. vesiculosus* (Sigma)	Human colon cancer HCT-15 cells	↑ ERK, p38 signaling ↓ PI3K/Akt signaling	[31]
	*U. pinnatifida*	Human lung cancer A549 cells	↑ ERK1/2 MAPK signaling ↓ p38, PI3K/Akt signaling	[42]
Inhibits angiogenesis and metastasis of tumor cells	*Sargassum hemiphyllum* (*S. hemiphyllum*)	Human hepatocellular carcinoma cells	↓ TGF- signaling	[43]
	*S.hemiphyllum*, Hi-Q Marine International Ltd.	human umbilical vascular endothelial cells (HUVECs) and human bladder cancer T24 cells	↓ HIF-1/VEGF	[44]
	*F. vesiculosus*	Lewis lung carcinoma cells	↓ NF-κB	[45]
	*S. hemiphyllum*	Human hepatocellular carcinoma cells	↓ miR-29b-DNMT3B-MTSS1 mediated TGF- signaling	[43]
	*U. pinnatifida sporophylls*	Mouse hepatocarcinoma Hca-F cell line	↓ HIF-1/VEGF-C ↓ PI3K/Akt/mTOR pathway	[46]
	*Laminaria cichorioides*	JB6 mouse epidermal cells	↓ JNK/c-Jun/AP-1 pathway	[47]
Synergistic effect with chemotherapy drugs	*F. evanescens*	Human T cell leukemia MT-4 cells	↑ Etoposide induced caspase-dependent cell death pathway	[48]
	*F. evanescens*	Lewis lung carcinoma bearing mice	↑ Anti-metastatic activity of cyclophosphamide	[49]
Enhanced immune function	*F. vesiculosus* (Sigma)	Lewis lung carcinoma (LLC) cells and mouse melanoma B16 cells	↑ Cytolytic activity of natural killer cells	[50]
	*F. vesiculosus* (Sigma)	NY-ESO-1 expressing human cancer cells	↑ Cross-presentation of NY-ESO-1 to T cells ↑ Cytotoxicity of T cells against NY-ESO-1-expressing human cancer cells	[51]
